# Automatic Detection and Quantification of WBCs and RBCs Using Iterative Structured Circle Detection Algorithm

**DOI:** 10.1155/2014/979302

**Published:** 2014-04-03

**Authors:** Yazan M. Alomari, Siti Norul Huda Sheikh Abdullah, Raja Zaharatul Azma, Khairuddin Omar

**Affiliations:** ^1^Pattern Recognition Research Group, Center for Artificial Intelligence Technology, Faculty of Information Science and Technology, Universiti Kebangsaan Malaysia, 43600 Bangi, Malaysia; ^2^Department of Pathology, UKM Medical Center, Universiti Kebangsaan Malaysia, Cheras, 56000 Kuala Lumpur, Malaysia

## Abstract

Segmentation and counting of blood cells are considered as an important step that helps to extract features to diagnose some specific diseases like malaria or leukemia. The manual counting of white blood cells (WBCs) and red blood cells (RBCs) in microscopic images is an extremely tedious, time consuming, and inaccurate process. Automatic analysis will allow hematologist experts to perform faster and more accurately. The proposed method uses an iterative structured circle detection algorithm for the segmentation and counting of WBCs and RBCs. The separation of WBCs from RBCs was achieved by thresholding, and specific preprocessing steps were developed for each cell type. Counting was performed for each image using the proposed method based on modified circle detection, which automatically counted the cells. Several modifications were made to the basic (RCD) algorithm to solve the initialization problem, detecting irregular circles (cells), selecting the optimal circle from the candidate circles, determining the number of iterations in a fully dynamic way to enhance algorithm detection, and running time. The validation method used to determine segmentation accuracy was a quantitative analysis that included Precision, Recall, and *F*-measurement tests. The average accuracy of the proposed method was 95.3% for RBCs and 98.4% for WBCs.

## 1. Introduction

The analysis of microscopy images is extremely important in both the medical and the computer science fields. Many research problems are related to the analysis of microscopy images, such as complete blood count (CBC) tests [1] and the analysis of blood smears, which is considered the first step in detecting and diagnosing malaria, leukemia, and anemia. Additionally, during a complete physical exam a series of tests are performed. One of these tests is the CBC, which is used to evaluate the composition and concentration of all cellular blood components. The CBC determines red blood cell (RBC) counts, white blood cell (WBC) counts, platelet counts, hemoglobin (HB) measurements, and mean red blood cell volumes [2].

CBC tests and the analysis of blood smear images help to evaluate, diagnose, and monitor various health conditions, such as anemia, leukemia, infections, and allergic conditions [3]. For blood disorders, such as anemia, which is based on HB level, the production and destruction of red blood cells are evaluated. In red blood cell disorder such as anemia, other red cell indices such as (mean cell volume) MCV, mean cell hemoglobin (MCH), mean corpuscular hemoglobin concentration (MCHC), RBC, and red blood cell distribution width (RDW or RCDW) are evaluated to narrow down on the causes of anemia. If the red cell indices are suggested of iron deficiency anemia (IDA), further tests to confirm the IDA will be done. In normal blood, red blood cell (RBC) counts range from 4.2 to 5.9 million cells per square centimeter. High RBC counts can be indicative of serious medical conditions, such as heart, lung, or kidney disease. Primary or secondary polycythemia in polycythemia HB is also raised; a bone marrow disorder also causes high RBC counts [2]. Normal WBC counts range from 4,500 to 10,000 WBCs per microliter of blood [4]. High WBC counts (above 30000 cells per microliter) indicate an infection, systemic illness, inflammation, allergy, leukemia, or burn-induced tissue injury. If leukemia is suspected, analysis of blood smear is done to look for morphology of the leukemic cells and followed by bone marrow examinations [2–4]. For platelets, which are small blood cell fragments that assist in blood clotting, normal counts range from 150,000 to 450,000 platelets per microliter. In patients with low platelet count such as in patients with dengue infection, their platelet count is monitored closely and the value is within critical level, the patient might need platelet transfusion [2]. Generally, any abnormal blood smear reading indicates an infection or disease.

Malaria and* Babesia* are parasites that infect RBCs; the analysis of thin blood smear remains the gold standard for diagnosis in such disease [5]. Microscopy images are also still used for early diagnosis, analysis, and count of some blood disorder such as sickle cell anemia and leukemia, before confirming it with other laboratory tests. However, manual or visual quantification of parasitemia in thin blood films and WBCs in leukemia is an extremely tedious, subjective, and time-consuming task with a high probability of counting error [6, 7]. An accurate segmentation and counting mechanism that gathers information about the distribution of microscopic particles may help diagnose abnormalities during clinical analysis. Our objective in this paper is to develop and validate an algorithm that segments and automatically counts red and white blood cells in microscopy images. The ground truth of the images was determined by experts. For the evaluation, quantitative analyses were performed on the segmentation results based on the ground truth, and the *F*-measure method was used to confirm accuracy. In the following sections of this paper, we will summarize related work on the segmentation and counting of RBCs and WBCs (Section 2), present the methodology used (Section 3), discuss the results and experiments (Section 4), and review the conclusions.

## 2. Related Work

Many researchers have investigated blood cell segmentation and counting. Some researchers [5, 8–10] used morphological operations and thresholding to do the segmentation and counting. Berge et al. [5] presented an approach based on a morphological method and iterative threshold techniques. Segmentation was performed on red blood cells, which included clumped cells, and boundary curvatures were used to construct a Delaunay triangulation. They used real microscopy images prepared in the laboratory, and the ground truth was determined by a laboratory expert. A 2.8% difference was calculated between the manual and automatic counting of red blood cells. Their method tolerated a degree of overlapping, but in cases with a high degree of overlapping cells, the cells were unable to be detected. Additionally, the iterative threshold method was unable to detect faint red cells. Damahe et al. [8] used the S and V image components of a HSV color model with Zack's thresholding technique for cell segmentation. Thresholding combined with a sequential edge-linking algorithm was used to increase segmentation accuracy. The experimental dataset and blood cell images were collected from the Dhruv Pathology Lab and the CDC-DPDx, respectively, and the ground truth was determined by experts. The dataset size was limited. Several RBCs that were detected possessed holes. Additional preprocessing steps should have been implemented to increase the accuracy. Panchbhai and Vishal [9] proposed an automated analysis that counted red blood cells and detected malaria parasites in thin blood smear samples. The green color layer was processed to count all of the RBCs; segmentation was performed on the infected RBCs using Otsu thresholding. A histogram was used to determine the optimal threshold. CDC datasets were used for the experimental part, and the ground truth was determined by a pathology expert who compared their results with the manual counts. However, because the detection algorithm used morphology and thresholding, their method was unable to detect clumped and overlapping cells. Khan et al. [10] proposed a method to count WBCs, RBCs, and platelets. Several preprocessing steps were performed before converting the image to binary. Segmentation and cell counting were performed based on the optimal threshold value, which was determined from a histogram. They achieved 95% accuracy with their proposed method compared to manual counting and a hematology analyzer. However, this method is unable to detect overlapping cells. When using iterative thresholds, the probability of losing useful information from the image is high; this decreases the accuracy of segmentation.

Nguyen et al. [11] used distance transform to solve the overlapping cells problem; they proposed a method that concentrated on clumped cells. First, they assigned central points based on a distance transform. The optimal center points were selected by checking the degree of boundary covering the center point, and the average size of a cell was estimated by the extraction of a single cell. Then an algorithm was developed that used a single cell mask to split the cells. Their dataset ground truth was labeled by experts, and the accuracy of the proposed method using *F*-measurements was 93.5% and 82%. Clumped cells were tolerated, but the cells had to be regularly shaped and focused at a high magnification. Not all blood cells have regular cell shapes, and this is especially true if the blood cells are diseased. Therefore, cell detection methods should be able to detect irregular cells. Additionally, because of noise the performance of their method was not good.

Rhodes and Bai [12] presented a circle detection method using specific properties of Gabor wavelet filter to detect the image features such as circularity. It is able to extract radius wraps around the origin and the plane wave radiates from the center of the filter. They test their proposed method on synthetic images and real microscopic images, which allow a certain degree of overlapping cells. The results were 91.3% and 87% of the cells detected in the two microscopic test images.

Since blood cells are approximately circular shape, circle detection algorithms can still handle the challenge of blood cells detection. Hough Transform is considered as one of the most known algorithms for line and circle detection. It was developed by Richard Duda and Peter Hart in 1972 [13]; they called it as a generalized Hough Transform after the related 1962 patent of Paul Hough [13]. Hough Transforms maps every edge pixel into parameter space and use conventional HT to detect lines, circles, or any other parametric shape. In this paper, we concentrate on circle detection algorithms. There are many techniques used for circle detection. Many HT-based algorithms that detect circles were developed using different methods. Yip et al. [14] reduced the accumulator array to enhance computation time and improve memory consumption. Other methods use pixel gradient information [15, 16] to reduce computation time and the accumulator array. Ho and Chen used the geometric properties of circles to improve performance [17]. Xu et al. [18, 19] developed the randomized Hough Transform (RHT), which randomly selects three noncollinear edge pixels (*a*, *b*, *r*), maps them into parameter space, and requires less computation time and memory storage compared with Standard HT methods. A simple voting strategy in the accumulator is used to collect evidence and determine the existence of a circle. Chung and Huang [13] presented a method that can substitute various shape detection algorithms. This method enhanced the speed of the original algorithm. They applied their method in RHT and randomized circle detection (RCD) algorithms to detect lines, circles, and ellipses. Their method presented good results when compared with original methods.

Chiu et al. [20] presented a fast randomized Hough Transform method for detecting circles, to improve RHT which is less efficient in complex images due to its probability usage problem. They pick one random edge pixel from the image, and it is considered as a seed point. Then, a checking method was developed to observe if this selected seed point is lying on a true circle or otherwise. The checking criterion is based on finding two other points whose distances are the same from the selected seed point by using a *W* × *W* window centered by the selected seed point. This method enhanced the probability to find relevant points on a true circle. They have proven that using one random selected point's probability is sufficient in comparison to three random selected points [13–19].

Chen and Chung [21] presented a circular algorithm called RCD. They claimed that RCD outperformed other most efficient Hough Transform based algorithms [13–19]. Regardless of accumulator usage as in [13–19], RCD works by randomly selecting four edge pixels from the whole image. Then, these pixels are examined if they are noncollinear and it will proceed to form a candidate circle. RCD determines that circle is a possible circle based on distance criteria. After finding its center and radius, it checks the number of pixels lying on the boundary of this possible circle. This checking criterion is performed by calculating the distance between all edge pixels in the image and the boundary of this possible circle. If this distance is lesser than a fixed threshold value, then it will be considered as a boundary of this possible circle. Finally, another fixed threshold value has also been used to decide a true circle or otherwise based on number of edge pixels lying on a possible circle's boundary. Other related issues on RCD are less efficient when dealing with huge image size consisting of a high number of edge pixels. RCD also requires four selected edge pixels randomly from the whole image, which causes low probability forming a true circle. Furthermore, RCD has a drawback in terms of its fixed number of iterations in which it is highly correlated to the image texture. In addition, RCD acquires many parameters and threshold values to be predefined and it ignores irregular circles.

Since the Hough Transform presented a good performance in different fields, many researchers [22–25] used Hough transform method for detecting circle when performing RBC's and WBC's calculation task in the microscopic images. Mahmood et al. [22] applied Hough transform method for counting the RBC's and WBC's for the microscopic images. In the first step, they converted the source image to *L*∗*a*∗*b* color space model and performed color segmentation process of red and white cells based on feature lightness over the *L* channel ranging between 130–150 and 80–100, respectively. Then, the second step begins with applying some morphological operators which are followed by applying Canny operator for edge detection process. Lastly, they performed cell detection and counting using Circular Hough Transform. The dataset was composed of 108 images from the “Acute Lymphoblastic Leukemia Image Database for Image Processing” database that was established and maintained by Labati et al. [26]. Depending on the processing and type of cells being analyzed, they achieved an accuracy ranging from 64% to 87%. The Hough Transform consumed memory storage and required a long computation time to determine a large range of accepted radii. Additionally, the ability to tolerate a high degree of overlapping and irregular cells was limited; therefore, accuracy was not high.

Mahmood and Mansor [23] examined 10 image samples of normal blood cells; image transformed to the HSV color space, and then Saturation or “S” channel was selected to proceed with image analysis. Morphological operators and thresholding method were used over S channel for cell segmentation. They used Circular Hough Transform to investigate the circularity feature of the red blood cells in order to perform detection and counting. Their proposed method achieved approximately 96% of accuracy rate in comparison to manual counting. Extracting and counting normal cells are simple tasks if the detected cells are normal cells and consist of small number of overlapping cells with regular shape. For this kind of easy case, the idea of using Hough Transform can be very helpful because it can produce a good performance.

With similar inspiration to [23], Venkatalakshmi and Thilagavathi [24] have also applied circular Hough Transform method to count the RBCs from microscopic images after performing preprocessing steps such as HSV transformation, S channel extraction, histogram thresholding morphological operations, XOR logical operation, and Canny edge detection. However, again, this proposed idea is less tolerant to any overlapping cells or irregular cells' shape. Later, Maitra et al. [25] presented a composition method to extract red cell from five microscopic images; these steps include spatial smoothing and filtering, adaptive histogram equalization, and edge detection. Similar to [23, 24], they used basic Circular Hough Transform to detect the red cells based on prior information such as size and shape features. Those methods [23–25] employ classic Hough Transform method to detect the blood cells which inherits some drawbacks, for example, required more computation, high memory consumption, and less ability in detecting overlapping cells or irregular cells.

Finally, Cuevas et al. [27] presented a method which is not Hough Transform based to detect white blood cells. They used a combination of circle detection with electromagnetism-like optimization from the edge map image. This method tolerated noise. In blood smear images, the number of white cells was small compared with the number of red cells; therefore, small degree of overlapping white cells can be detected with their method. However, they did not test the method on clumping cells. Their results were compared with other methods, and their method demonstrated good accuracy.

## 3. Methodology 

The proposed method was developed to analyze microscopic images of blood smears by segmenting and counting both WBCs and RBCs. The segmentation is based on thresholding and morphological operations, and then counting is based on the circularity feature of the blood cells extracted using an iterative structured circle detection algorithm.

**Figure 1:**
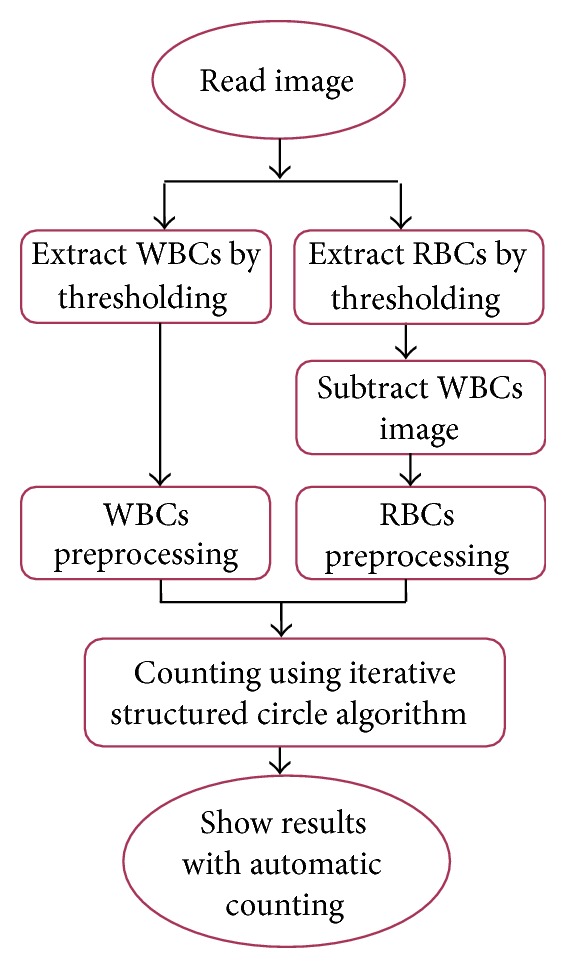
General methodology for the proposed method.

A new technique for binary images based on the fundamentals of RCD has been proposed and used for counting RBCs and WBCs. Therefore, the original image is separated into two images; the first image contains RBCs only and the second image contains WBCs; this step has been done using thresholding. We study the histogram for 20 sample gray scale images, and we find out the best thresholding values to extract WBCs and RBCs; values were 64 and 140, respectively. After cells separation, each image is preprocessed using morphology operators to obtain the edge image using Canny operator. Then, an iterative structured circle detection algorithm is used to count cells in each image.  Figure 1 shows the proposed method for WBC and RBC segmentation.

### 3.1. Preprocessing

The proposed method for cell segmentation works with edge images. Microscopy images of blood smears are colored images, and several steps are required to prepare the image before extraction of the edge image. In our proposed method, the cells were separated by type and distinct preprocessing steps were developed for WBCs and RBCs separately.

*Preprocessing for WBCs.* At this stage, white blood cells are extracted as a separate image, and the red blood cells have varying intensities; therefore, it is preferable to develop separate preprocessing steps for each cell type.

**Figure 2:**
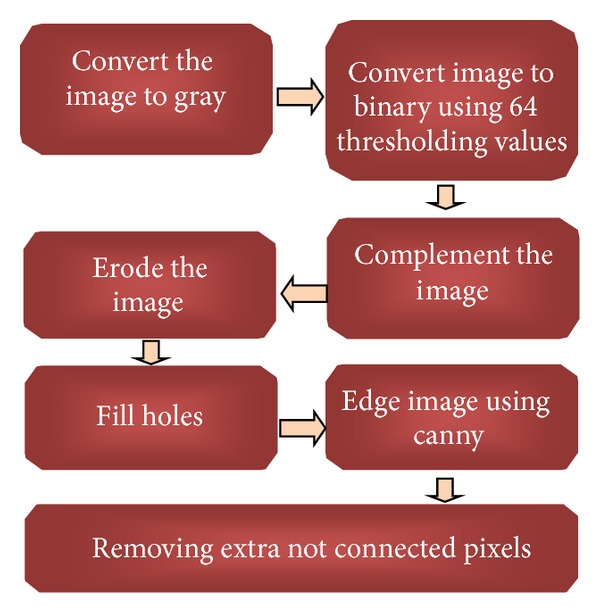
Preprocessing steps for WBCs.

**Figure 3:**
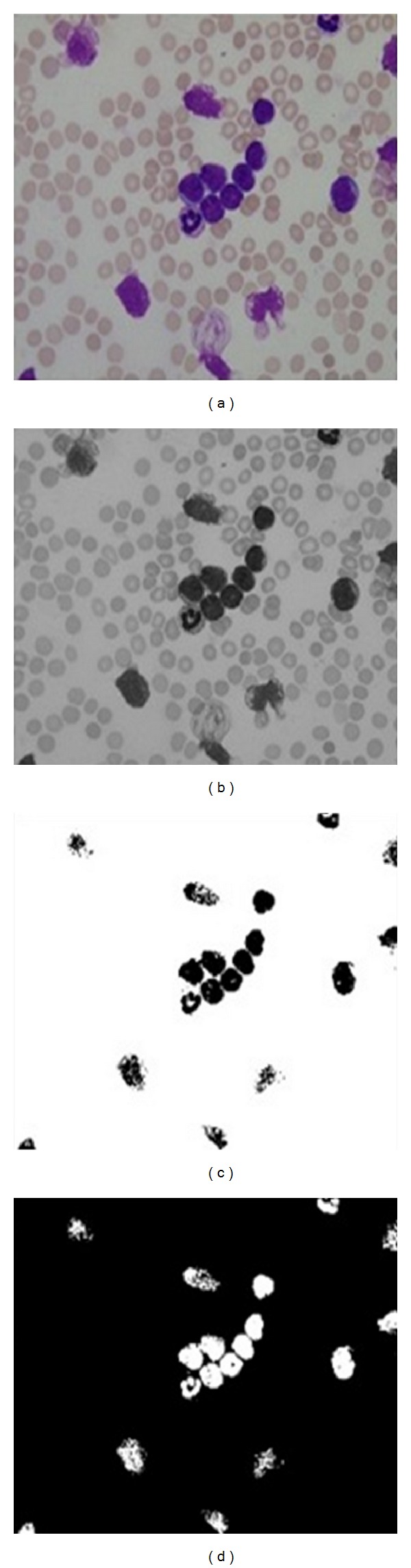
(a) Original blood image, (b) gray image, (c) binary image using thresholding, and (d) complement image.

Figure 2 shows the overall preprocessing steps for WBCs. To remove RBCs from the image, the RGB image was converted into grayscale image by eliminating the Hue and saturation information while retaining its luminance, as shown in Figure 3(b), and then converted the image to binary using thresholding using a threshold value (64); visualize the WBCs, as shown in Figure 3(c). Some undesired holes appeared in the cells, and the morphology operator, fill holes were used to remove them. The complementary images before the holes were filled, as shown in Figure 3(d).

**Figure 4:**
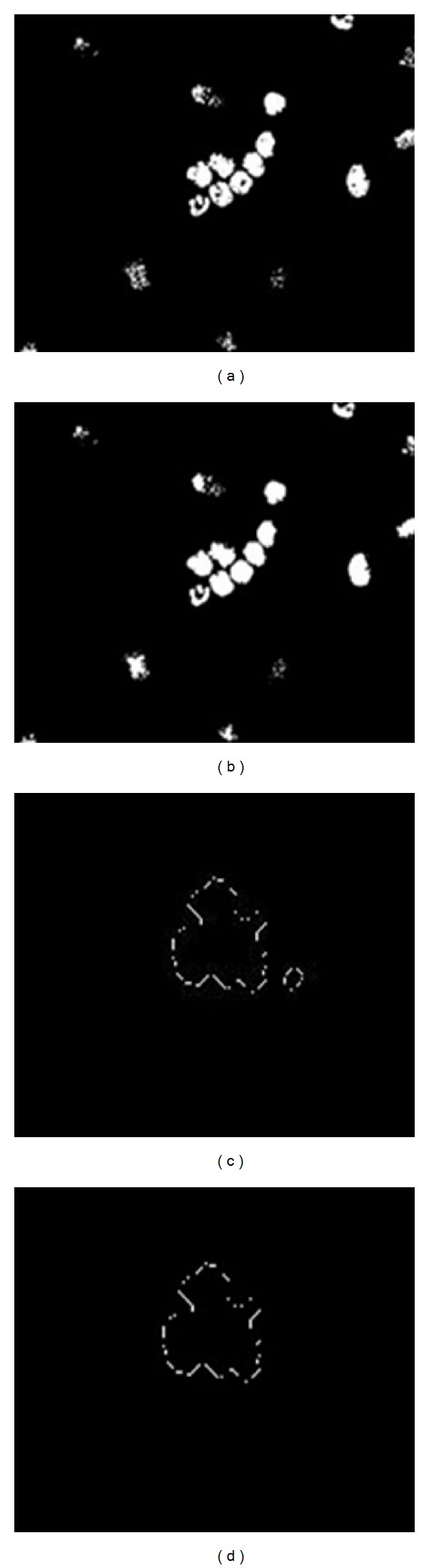
(a) Eroded image, (b) image after filling holes, (c) edge detection using a Canny at high magnification, and (d) image after removing the noise at high magnification.

This image eroded to reduce the number of overlapping cells, as shown in Figure 4(a). Because the boundary of the cells is required, the holes were filled to improve the edge detection.  Figure 4(b) shows the image after the holes filled. The Canny operator was used to visualize the cell edges, as shown in Figure 4(c). After edge detection, some undesired pixels appeared that affect the segmentation process. These pixels were removed using an open morphology operator, as shown in Figure 4(d).

#### 3.1.1. Preprocessing for RBCs

**Figure 5:**
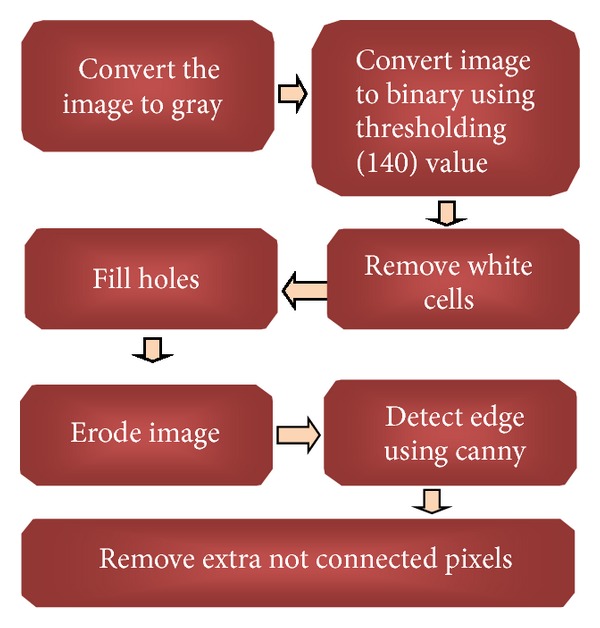
Preprocessing steps for RBCs.

In this stage, preprocessing was performed on the red blood cells after removing the white blood cells from the image.  Figure 5 shows the overall preprocessing steps for the RBCs. First, the original image is converted into grayscale image by eliminating the Hue and saturation information while retaining its luminance.

**Figure 6:**
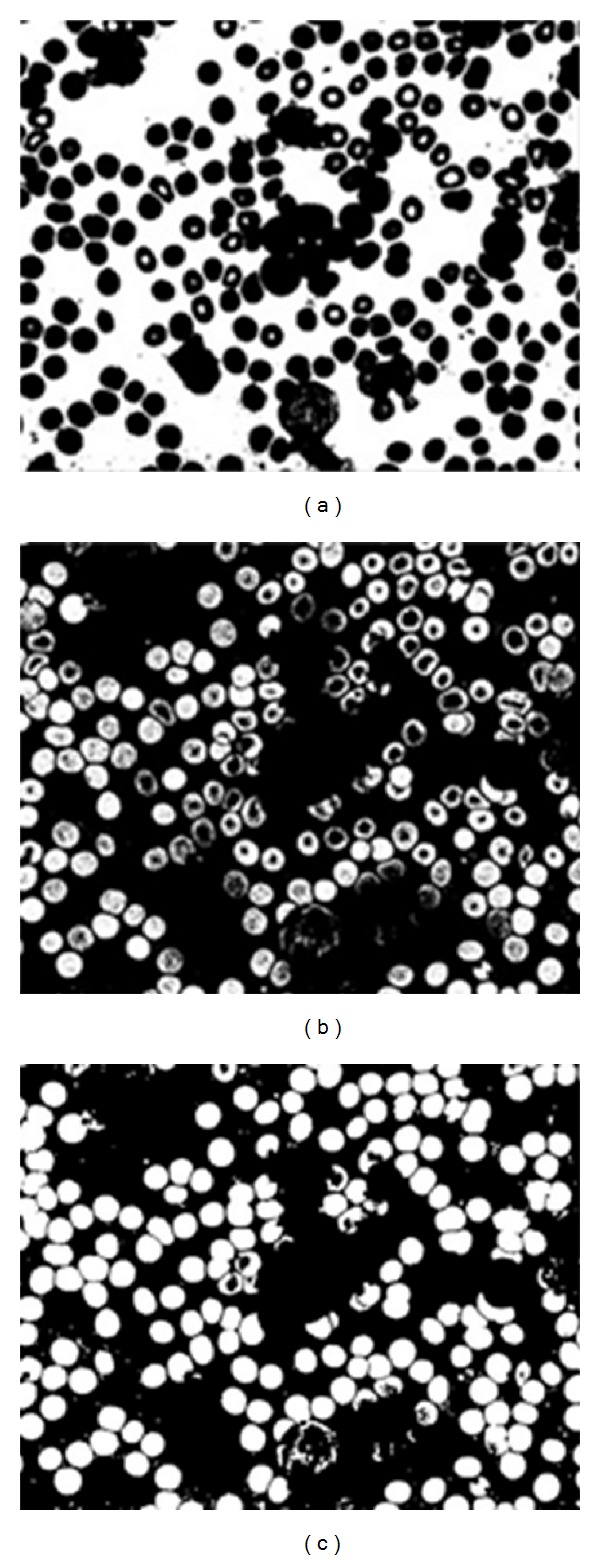
(a) Binary image using thresholding, (b) RBCs after removing the WBC's by subtracting the two images, and (c) image after filling holes.

Then, image was converted to binary using thresholding value of (140), to visualize all of the red and white blood cells in the image, as shown in Figure 6(a). To remove the white blood cells from the image, the complementary white cells image was taken and subtracted from the first image to obtain only the red cells, as shown in Figure 6(b).

When the image was converted to binary and the white blood cells were removed, undesired holes were created. These holes disturb the solidness of the object. Therefore, morphological operators are used to fill the holes, as shown in Figure 6(c).

**Figure 7:**
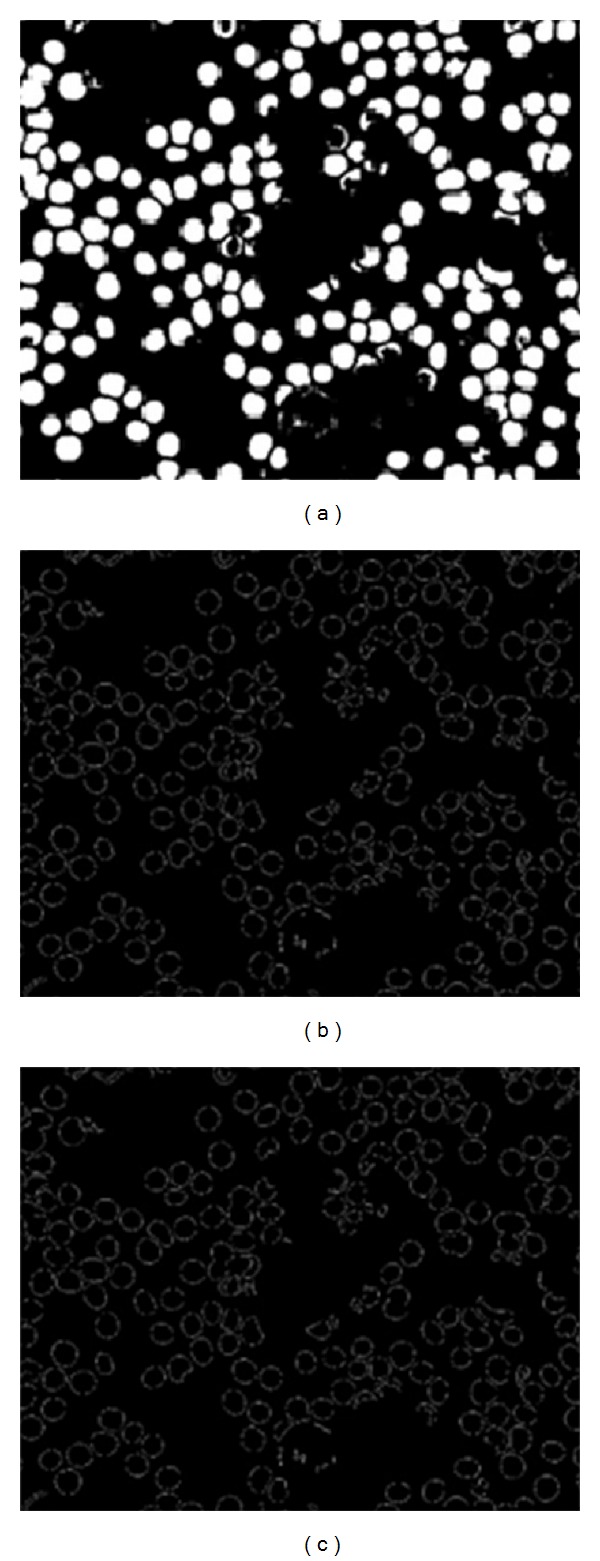
(a) The eroded image, (b) image after edge detection using thresholding, and (c) after removing extra unconnected pixels.

A morphological step using an erosion operator is used to reduce the overlap between cells, as shown in Figure 7(a). Canny edge detection method is used to obtain the edge image, as shown in Figure 7(b). Some undesired pixels appeared after edge detection. These pixels represent either platelets or noise, and it will subsequently affect the segmentation process. Therefore, these pixels are removed using a morphology operator on the binary image, as shown in Figure 7(c). After these preprocessing steps, the image was prepared for input to our proposed circle detection algorithm.

### 3.2. The Proposed Method for Counting

**Figure 8:**
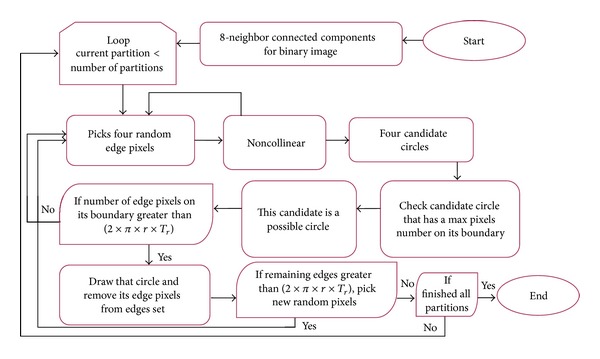
Proposed method workflow.

The basic idea for the proposed method was derived from the RCD [21] algorithm. RCD algorithm ignores accumulator capability that has been introduced by Hough Transform method. Several modifications were made to the basic RCD algorithm to solve the initialization problem when using big images with high number of pixels. The methods modified to detecting irregular circles, selecting the optimal circle from the candidate circles, determining the number iterations in a fully dynamic way to enhance the algorithm detection and running time, and improving the detection of overlapping cells. The workflow of the proposed method is shown in Figure 8.

*Initialization Problem.* The proposed method partitions the edge image based on 8-neighbor connected components in order to overcome the initialization problem caused in basic RCD [21]. We eventually divide the whole image into small partitions and we consider each partition as an input image before entering into our iterative structured circle detection algorithm that employs local randomization step.

**Figure 9:**
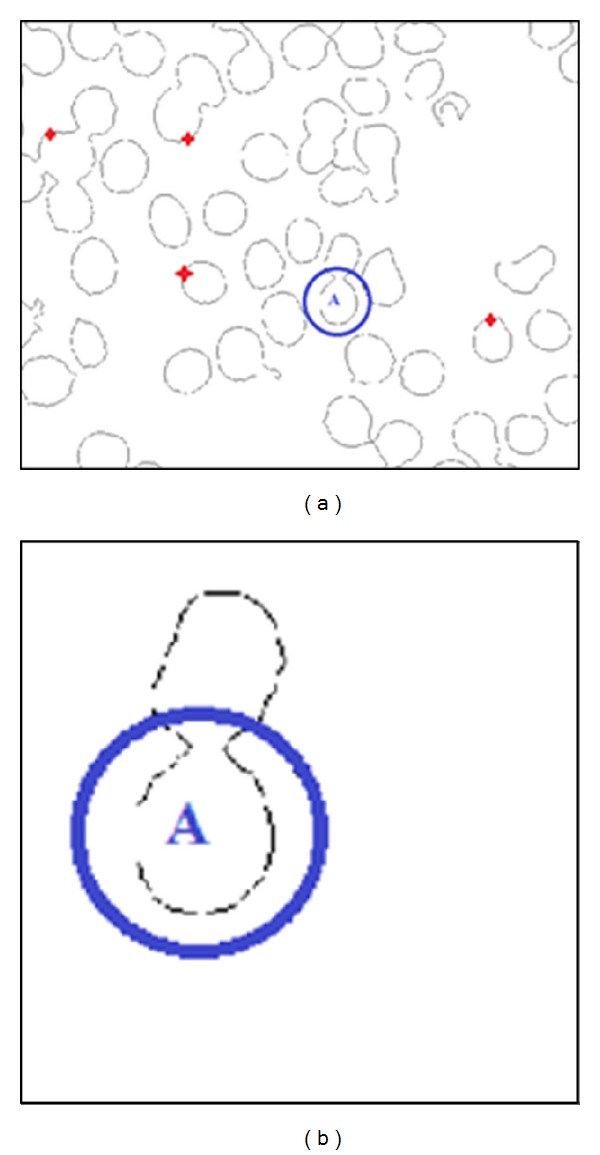
(a) Initialization problem in basic RCD in real image, and (b) partition from an image.

Regarding to this initialization problem, as we highlighted earlier that selecting four pixels globally (from the whole image) can reduce the probability of finding true circle, time consuming, and needs of a high number of iterations to find all true circles. This can be easily illustrated as in Figure 9(a) if cell A is to be detected, and the four selected pixels are chosen in Red Cross mark. Therefore, we simplify this process by introducing four pixel selections locally (within each partition image). Assume that cell A is to be detected in the partition image, and then these four local pixels are chosen randomly from the partition image as depicted in Figure 9(b). This local randomization process repeats until all partition images are visited.

In each partition image, our proposed method randomly selects up to four edge pixels and checks the existence of a circle using (1), which is based on three noncollinear pixels (v1, v2, and v3):

(1) $$\begin{array}{c} \left(x_{j}-x_{i}\right)\left(y_{k}-y_{i}\right)-\left(x_{k}-x_{i}\right)\left(y_{j}-y_{i}\right)=0. \end{array}$$

If the result is zero, the three-edge pixels are collinear and cannot form a circle; they are returned to the edges array for the chosen partition, and four new subsequent pixels are selected from the same partition.

**Figure 10:**
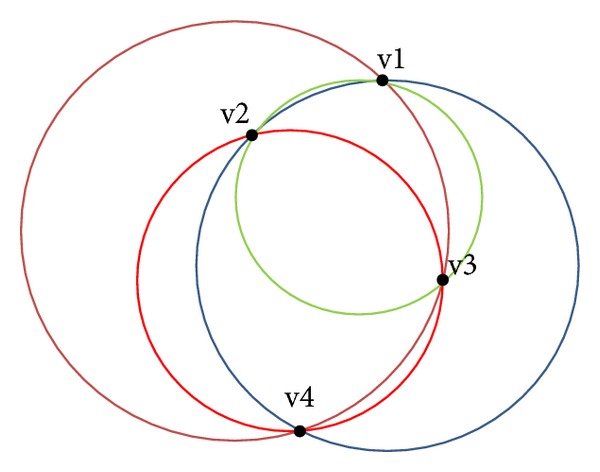
Four candidate circles formed using four edge pixels.

If the pixels are not collinear, they are able to form a circle. Four candidate circles, C_123_, C_124_, C_134_, and C_234_, can be formed using the four edge pixels (v1, v2, v3, and v4), as shown in Figure 10. Each circle is formed from three edge pixels, and the center (*a*, *b*) and radii (*r*) were calculated using (2), (3), and (4), respectively. Consider the following:

(2) $$\begin{array}{c} a_{ijk}=\frac{\left|\begin{array}{cc} x_{j}^{2}+y_{j}^{2}-\left(x_{i}^{2}+y_{i}^{2}\right) & 2\left(y_{j}-y_{i}\right)\\ x_{k}^{2}+y_{k}^{2}-\left(x_{k}^{2}+y_{i}^{2}\right) & 2\left(y_{k}-y_{i}\right) \end{array}\right|}{4\left(\left(x_{j}-x_{i}\right)\left(y_{k}-y_{i}\right)-\left(x_{k}-x_{i}\right)\left(y_{j}-y_{i}\right)\right)}, \end{array}$$

(3) $$\begin{array}{c} b_{ijk}=\frac{\left|\begin{array}{cc} 2\left(x_{j}-x_{i}\right) & x_{j}^{2}+y_{j}^{2}-\left(x_{i}^{2}+y_{i}^{2}\right)\\ 2\left(x_{k}-x_{i}\right) & x_{k}^{2}+y_{k}^{2}-\left(x_{i}^{2}+y_{i}^{2}\right) \end{array}\right|}{4\left(\left(x_{j}-x_{i}\right)\left(y_{k}-y_{i}\right)-\left(x_{k}-x_{i}\right)\left(y_{j}-y_{i}\right)\right)}, \end{array}$$

(4) $$\begin{array}{c} r_{ijk}=\sqrt{{\left(x_{i}-a_{ijk}\right)}^{2}+{\left(y_{i}-b_{ijk}\right)}^{2}}. \end{array}$$

In basic RCD algorithm, circle can be formed with three prior pixels; the fourth pixel is used to obtain four candidate circles at a time for each iteration, which is better than forming one candidate circle for each iteration. However, in our proposed method, one of the candidate circles, C_123_, C_124_, C_134_, and C_234_, is selected to be possible circle. This based on the candidate with the highest probability of being a possible circle. This can be achieved by checking the number of edge pixels located on the boundary of each candidate circle. We assumed that candidate circle with the maximum number of edge pixels on its boundary is considered as a possible circle. The following calculates the distance between each edge pixel (*z*) in the partition and the boundary of the candidate circle:

(5) $$\begin{array}{c} d_{z\to ijk}=\;\left|\sqrt{{\left(x_{z}-a_{ijk}\right)}^{2}+{\left(y_{z}-b_{ijk}\right)}^{2}}-r_{ijk}\right|. \end{array}$$

After checking all of the edge pixels in the partition and selecting the possible circle, the number of edge pixels located on its boundary is determined. If this number is greater than the value obtained from  (2 × *π* × *r* × *T*_*r*_), then the possible circle is considered as a real circle. Where *T*_*r*_ = 0.7 for WBCs and *T*_*r*_ = 0.5 for RBCs. (2 × *π* × *r*)  is the circle perimeter, and this value should be equal to the estimated number of pixels on the circle boundary. *T*_*r*_ is the accepted ratio of the total number of pixels on the possible circle boundary that determines that the circle is a real circle. Therefore, if 70% or 50% of edge pixels are located on the boundary of possible circle for WBCs or RBCs, respectively, the possible circle is considered a real circle. Then the edge pixels are removed from the set of edges in that partition and the process starts again to find other circles within the same partition. If there are not enough remaining edge pixels or less than the threshold value of *T*_min⁡_ = (2 × *π* × *r*_min⁡_ × *T*_*r*_), where *r* is the minimum radius possible for red or white blood cells and *T*_*r*_ is one of the values mentioned above, then the next partition is selected and the process begins again. Unlike basic RCD, our proposed method applies dynamic *T*_min⁡_ thresholding value which depends on minimum acceptable radius (*r*_min⁡_) value and *T*_*r*_ value pertaining to the type of cells to be detected.

If the number of edge pixels on the boundary of the possible circle is less than the threshold value *T*_*r*_, four new edge pixels are randomly selected. The *T*_min⁡_ value is determined based on the minimum number of pixels that can form a circle boundary with a radius *r*  and is estimated to be (2 × *π* × *r* × *T*_*r*_) with a width of one pixel.

By adding the 8-neighbor connected component step, the algorithm is guaranteed to randomly select edge pixels from every part of the image, check the circle possibilities for all image edge pixels, and locate more true circles, which improves the performance of the method, especially for large images with a high number of pixels.

**Figure 11:**
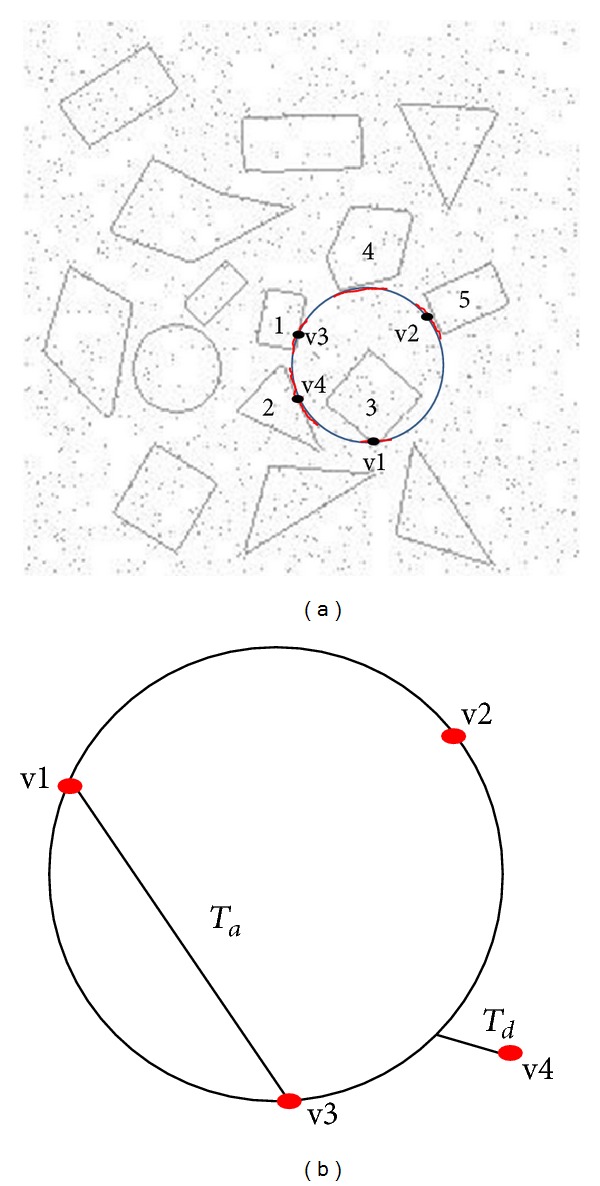
(a) Wrong circle detection in basic RCD algorithm and (b) some basic RCD thresholding values.

Another drawback of basic RCD algorithm, it chooses four random edge pixels from the entire or global image that may cause chosen pixels from different actual shapes as depicted in Figure 11(a). These edge pixels can form a circle based on three threshold values (*T*_*a*_, *T*_*d*_, and *T*_*r*_); Figure 11(b) shows some basic RCD threshold values. *T*_*a*_ represents the threshold value of distance between randomly selected edge pixels; *T*_*d*_ represents the threshold value of distance between the fourth edge pixel and the boundary of the possible circle, and *T*_*r*_ threshold value of accepted ratio of the number of pixels on the possible circle boundary, as (6). Based on these threshold values, basic RCD decided that this circle is a true circle, even if it is untrue. Figure 11(a) demonstrates how the basic RCD algorithm selects four pixels randomly from the numbered shapes (1, 2, 3, and 5) and forms a possible circle based on *T*_*a*_, *T*_*d*_, and *T*_*r*_ thresholding values:

(6) Cijk={accept  as  a  possible  circle if  (d(ij)∩d(ik)∩d(jk)>Ta)  ∩(dz→Cijk<Td)accept  as  a  true  circle elseif  Number  of  edge  pixels  on⁡ Cijk  boundary>Trchoose  another  random  pixels else.

Taking into account the above problems, we introduce partition based on 8-nieghbor connected components in our proposed method. We restrict up to four random selected pixels from a particular partition. Next, we also check the number of edge pixels located on the possible circle boundary within that particular partition. Having known these pieces of information, it guarantees (1) better improvement performance and (2) higher probability of finding all existed true circles in that source image and reduces true-negative circles.

**Figure 12:**
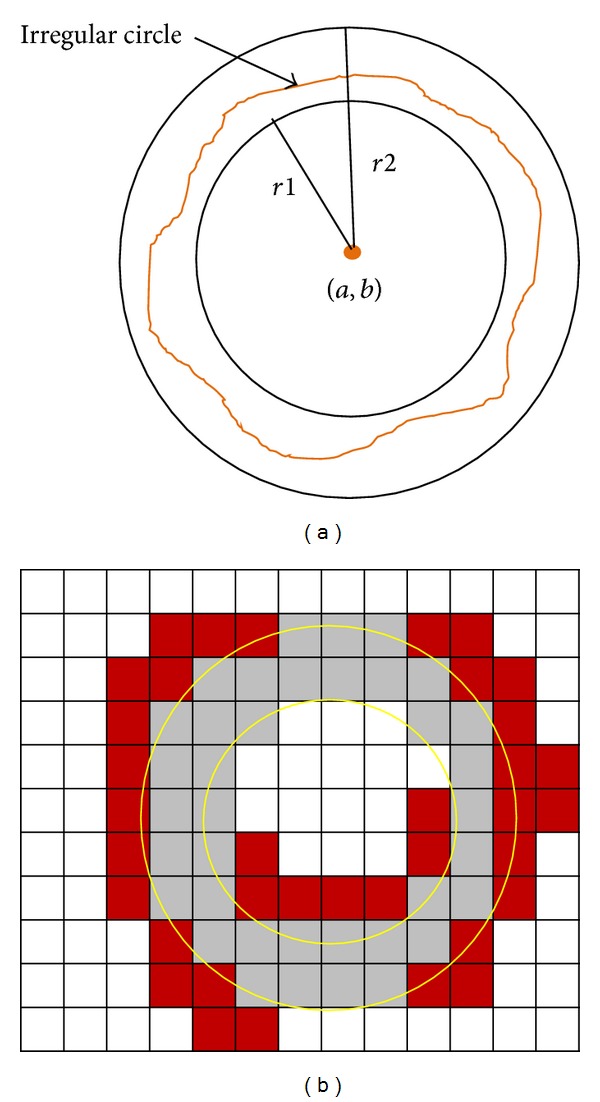
(a) The irregular circle detection method and (b) the detection of gray pixels of the irregular cell.

*Irregular Circle Detection.* Normal red and white blood cells are approximately circular; however, not all blood cells are regular circles. Therefore, we detected irregular circles whenever a possible circle was found. We defined a virtual circle with the same center and different radii, as shown in Figure 12(a). When the algorithm locates a possible circle, we superimpose these two circles on the grid and check all of the edge pixels in the segment and detect all edges lying between the two circles.  Figure 12(b) shows the irregular circle pixels, which are located between the two virtual circles in gray color are detected.

The red pixels are not included in the boundary of candidate circle. By partitioning the image based on connected components, we increase the distance between the two circles. This ensures that the detection edges on the boundary of the possible circle will be correct without using edges from other shapes, as observed in Figure 11(a).

*Our Proposed Iterative Structured.* The number of iterations is extremely important to find all circles in the partition image. Therefore, determining the number of iterations is not an easy task for several reasons. Firstly, the number of iterations predominately depends on two factors: the number of edge pixels in the image and the distance between the edge pixels that are required to form a circle in each iteration. As a result, this may cause huge numbers of iteration to find all possible circles in the image. Our proposed method suggests a dynamic way to decide number of iterations for each partition image. We introduce two other factors: the number of pixels in the partition image and the cell radius.

**Figure 13:**
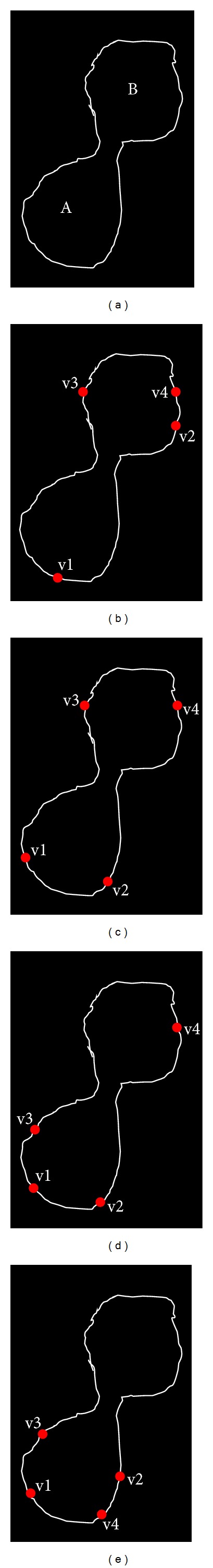
Example of (a) partition image, (b) first, (c) second (d), and third random pixel distribution cases demonstrating unacceptable circles and (e) the accepted case of random pixel distribution.

For example, assume we have a partition image, as shown Figure 13(a); it has 1000 edge pixels, and we search for the circle with a minimum circumference or perimeter value (2 × *π* × *r* × 0.7). If *r* = 100 and the perimeter has a width of one pixel size, then it is about 440 edge pixels can be formed as a circle perimeter (2 × *π* × 100 × 0.7). Therefore, we can assume that Figure 13(a) contains at most (2 ≈ 1000/440) circles.

According to the information above, the number of iterations can be determined as detailed below. Suppose random pixels were selected on the first iteration, such as in Figure 13(b). This case cannot represent the accepted circle because it does not meet with the proposed method threshold value *T*_*d*_ and *r*_max⁡_ parameter in order to detect any of the two cells (A, B). If v2, v3, and v4 form a circle as in Figure 13(b), then the proposed method rejects that candidate circle because the distance between v1 and the circle is greater than threshold value *T*_*d*_ and the candidate circle's radius exceeds *r*_max⁡_. This will be similar to cases in Figures 13(c) and 13(d).

**Figure 14:**
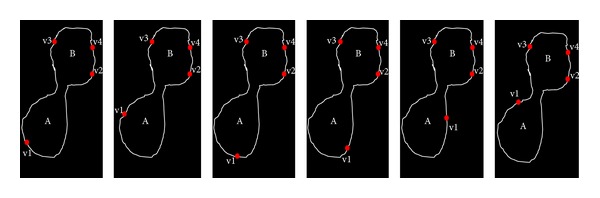
Some cases for unacceptable circles for the case shown in Figure 13(b).

Figure 14 explains in detail the subsequent processes as shown in Figure 13(b). Assuming that there are approximately 500 edge pixels forming each cell (A and B), and the cell (A) is to be detected; then about 500 number of iterations are required to find all unacceptable circles for each distribution of random pixels as shown in Figures 13(c) and 13(d). This is due to found circles that do not meet requirement of *T*_*d*_ threshold and *r*_max⁡_ parameter values. Therefore, the total number of iterations for this partition image may arise up to 1500. In other word, 500 multiply with 3 cases as shown in Figures 13(b), 13(c), and 13(d). The only acceptable case is shown in Figure 13(e). Here, the random pixel distribution meets our proposed method's condition dealing with *T*_*d*_ thresholding and *r*_max⁡_ parameter values in relation to accepted circle formation. Based on the previous case, the proposed method requires 1500 iterations at most to find all of the unaccepted circles, and the accepted circle should be located by iteration 1501.

Because the proposed method requires a certain distance between random edge pixels on the same circle, the number of iterations was increased slightly to tolerate the proposed method conditions and ensure that all possibilities are included. The final expression for the number of iterations is as follows:

(7) Iterations  Number=(Partition  Edge  Pixels  Number  ×(Number  of  Randomly  Edge  Pixels  Selected−1)).

For the previous example, the proposed method requires (1000 × (4 − 1)) = 3000 iterations to locate all of the circles in Figure 13(a). For this case, there is no fixed number of iterations for any of the partitions. The number of iterations is fully dynamic based on the relation between the number of edge pixels in the partition and cell radius.

### 3.3. Steps of the Proposed Method

**Step 1**

Divide the entire binary image to a small partitions based on 8-neighbor connected components. Each partition is denoted by *P*_*i*_ so that each image is defined by the set *P*_1_, *P*_2_,…, *P*_*n*_, where *P* is the number of edge pixels for each partition, *n* is the number of partitions, and *f* is the number of failures that can be tolerated. The failure counter is initialized so that *f*  is set to 0.

**Step 2**

The algorithm loops from *P*_1_ to *P*_*n*_.

**Step 3**

If |*P*_*i*_| ≤ *T*_min⁡_ or *f* ≥ ((*v* − 1) × |*P*_*i*_|), where *v* is the number of randomly selected pixels and *T*_min⁡_ is the minimum number of pixels allowed to start the method, the algorithm proceeds to Step 2; otherwise four edge pixels,  *v*1,  *v*2,  *v*3, and  *v*4, are randomly selected and removed from *P*_*i*_.

**Step 4**

**Figure 15:**
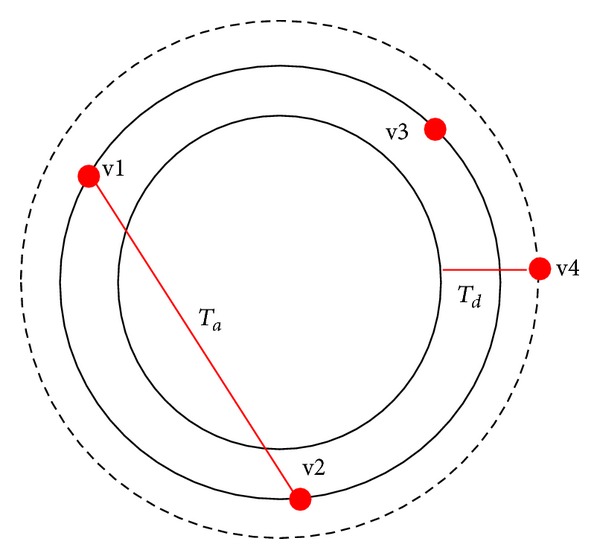
Proposed method parameters.

Determine the four candidate circles from the four edge pixels such that the distance between any two of the three pixels that forms the circle is greater than *T*_*a*_, *T*_*d*_ the distance between the possible circle and virtual circle. The fourth pixel must come between the two circles; the distance between fourth edge pixel and the boundary of the virtual circle is less than *T*_*d*_.  Figure 15 shows the proposed method thresholding values and parameters, and the radius  *r*_min⁡_ ≤ *r* ≤ *r*_max⁡_. If this is true, the algorithm proceeds to Step 5; otherwise the four edge pixels are returned to *P*_*i*_. Then, *f* = *f* + 1 and the algorithm continues to Step 3.

**Step 5**

For each candidate circle, the number of pixels on the boundary is calculated using (5) and the circle with the maximum number of pixels on its boundary is assumed a possible circle because it has the highest probability.

**Step 6**

If the number of pixels on the boundary of the possible circle is greater than (2*πrT*_*r*_). Where *T*_*r*_ is a threshold value equal to 0.5 for RBCs and 0.7 for WBCs. Then it is a real circle and all of the pixels found on its boundary are removed from the set of pixels *P*_*i*_, and *f* is set to 0. The algorithm returns to Step 3 to find other circles. Otherwise this possible circle is false, *f* = *f* + 1, and the algorithm returns to Step 3.

The proposed method has a few thresholding values (*T*_*a*_, *T*_*d*_, and *T*_min⁡_) and parameters (*r*_min⁡_ and *r*_max⁡_) to be determined because they have direct correlation on the detected cell results. For RBC's and WBC's, the radiuses of [*r*_min⁡_, *r*_max⁡_] were measured using simple distance measure ranging from [10, 50] and [20, 70] pixels, respectively. This helps to increase both the probability of detecting correct cells and performance of overlapping cells.

Specifying *T*_min⁡_ thresholding with the value (2 × *π* × *r*_min⁡_ × *T*_*r*_), it improves the proposed method performance. It stops searching for a circle when the number of edge pixels in the partition is less than *T*_min⁡_. *T*_*a*_ thresholding values also influence the proposed method performance. As *T*_*a*_ value decreases, both number of candidate circles and probability of detecting wrong circles are increasing (FP); otherwise the number of detected circles is reducing. Apart from that the *T*_*d*_ thresholding value represents the distance between the two virtual circles and this condition helps our proposed method to detect irregular circles (*T*_*d*_ = 20) and increases the number of edge pixels to be included on the boundary of the possible circle.

As conclusion, several improvements were made to the basic RCD algorithm, including the initialization step, solved by addition of 8-neighbor connected component step. Which divide the image into smaller partitions and the ability to select random edge pixels locally from the smaller parts instead of choosing random edge pixels globally from the entire image. This increased the probability of locating more circles. Not all blood cells have a regular circular shape. By increasing the thickness of the circle perimeter, the proposed method is also able to detect irregular cells. Partitioning the image and specifying the cell radius in the proposed model improved the detection of overlapping cells. Finally, we determined a relationship between the cell radius and the number of edge pixels in the partitioned image to control the number of iterations.

## 4. Experimental Results and Discussion

### 4.1. Dataset

The dataset used in this paper consisted of 100 actual microscopy images of blood samples. The images captured with an optical laboratory microscope coupled with a Canon Power Shot G5 camera. All of the images are in JPG format with 24-bit color depth and a resolution of 2592 × 1944 pixels. The images were taken at different microscope magnifications ranging from 300 to 500x [26].

### 4.2. Ground Truth

The ground truth was determined by an expert and used to validate our proposed method results.

### 4.3. Evaluation Methods

The results from the proposed method were quantitatively analyzed based on the ground truth. Precision, Recall, and *F*-measurements were used to determine segmentation and counting accuracy with the following equations:

(4.3) $$\begin{array}{c} \text{PR}=\frac{\text{TP}}{\left(\text{TP}+\text{FP}\right)},\\ \text{RC}=\frac{\text{TP}}{\left(\text{TP}+\text{FN}\right)},\\ F\text{-Measure}=\frac{2}{\left(1/\text{PR}+1/\text{RC}\right)}. \end{array}$$

We divided the dataset into 10 sets. For each set, we determined the average true positive value (TP), which is when the agreement between the expert and the proposed method for the detected cells. The false negative (FN), which is when the proposed method was unable to detect the cell but the expert detected a cell, and the false positive (FP), which is when the proposed method detected a cell but the expert did not. Then, we calculated the Precision (PR), Recall (RC), and *F*-measures (FM) of each set. The overall PR, RC, and *F*-measures for the proposed method are listed on the far right in Tables 1 and 2.

Table 1 summarizes the segmentation and counting accuracy of the proposed method for WBCs. The following segmentation and counting accuracies were calculated using the proposed method for WBCs: PR = 90%, RC = 98%, and *F*-measure = 94%.

Table 2 summarizes the segmentation and counting accuracy results of the proposed method for RBCs. The following is the segmentation and counting accuracies of RBCs that were calculated using our proposed method: PR = 95%, RC = 98%, and *F*-measure = 96%.

**Figure 16:**
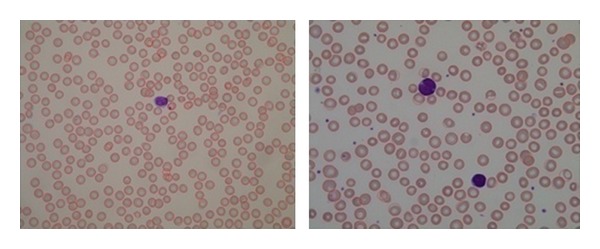
RBCs detected by the proposed method.

**Figure 17:**
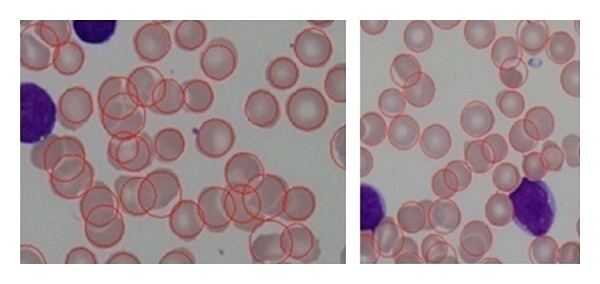
Overlapping cells detected by the proposed method.

Figure 16 shows sample images from the dataset and RBCs detected using the proposed method. These samples did not have many overlapping cells. Our proposed method was able to tolerate a variable degree of overlapping cells.  Figure 17 shows sample images from the dataset that contain overlapping cells and the RBCs that were detected using the proposed method.

**Figure 18:**
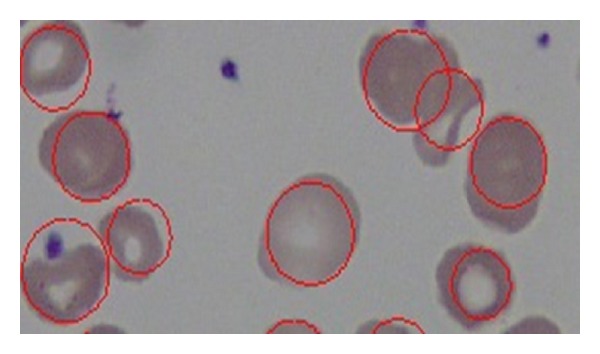
Real case example of irregular cells detected.

Additionally, our proposed method was able to perform for cases with irregularly shaped cells.  Figure 18 shows an example of the irregularly shaped cells that were detected using the proposed method. The RBCs had irregular shapes and were detected with the proposed method.

**Figure 19:**
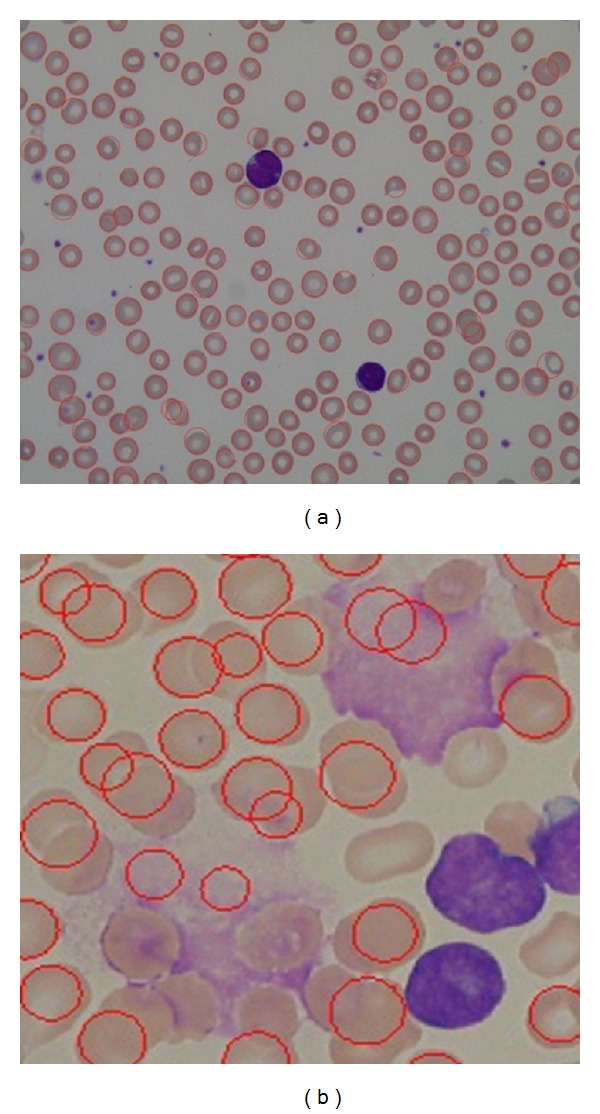
(a) Sample from our results for normal cases and (b) staining problem in some cases.

**Figure 20:**
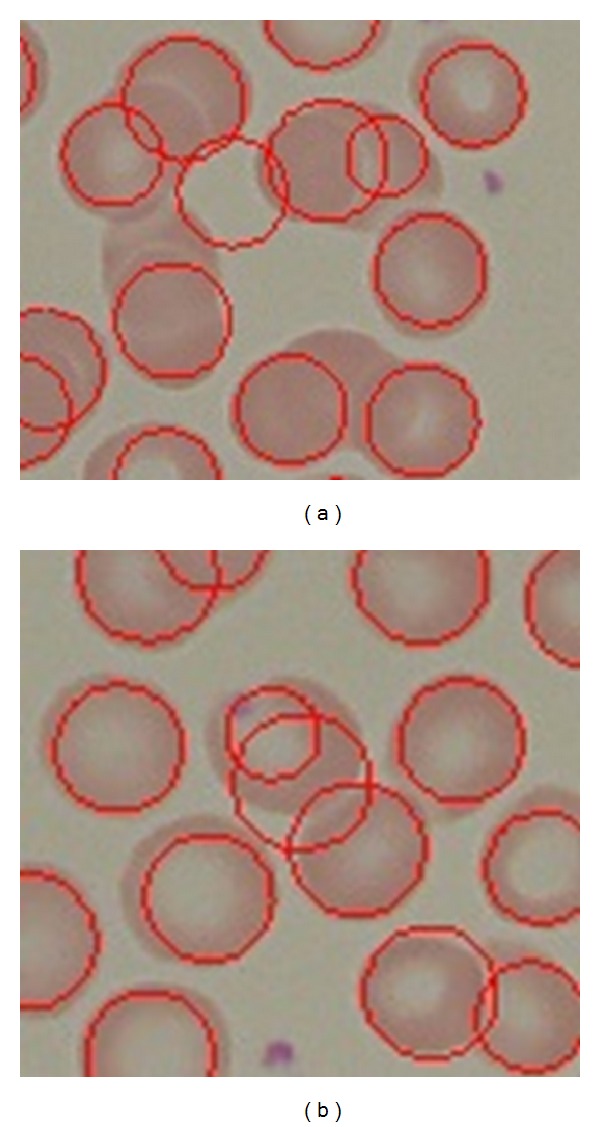
(a) Wrong detection in some case from our proposed method and (b) preprocessing problem.

Tables 1 and 2 show some testing groups presenting a quite high FP rate. This is due to microscopic images used for Leukemia blood cells patients. These images in some cases (healthy cases) and the cells are not crowded and clumped together and our proposed method's performance was acceptable with low FP rate as shown in Figure 19(a). In some other cases, some images have problems in staining as shown in Figure 19(b). Unfortunately, our proposed method has detected some true negative cells. It finds random edge pixels that can form a circle and the number of edges on the boundary of the possible circle is adequate; however, this leads to detect the wrong circle as shown in Figure 20(a). In some other cases, error in preprocessing steps such as incorrect holes filling problem creates negative effect on edge detection phase as shown in Figure 20(b).

**Figure 21:**
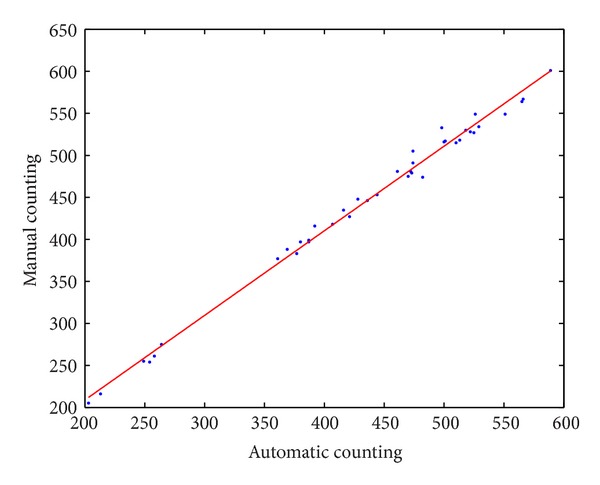
The outliers have no influence on our proposed result as it consistently fits in a regression line.

We evaluate our proposed method's result using a simple least square method in RANSAC algorithm. We take 40 random samples from our automatic and its corresponding manual counting results and apply them on the RANSAC algorithm. Our findings show that the selected results consistently fit a regression line as shown in Figure 21. Therefore, our data can be classified as a consensus, which means that our results are reasonably acceptable because most of the points have been classified as part of the consensus set.

### 4.4. Comparison of the Results with Other Methods

The average accuracy of the proposed method was 97.5% for RBCs and 98.4% for WBCs. We compared our proposed method with the method presented in [22]. They used the same dataset, performed segmentation, and counted WBCs and RBCs. They used color-based segmentation using *L*∗*a*∗*b* color space and the Hough Transform for cell extraction and counting. The results for all of the images were based on a user specified CIELAB range. The accuracy of their results ranged from 64% to 87%. In our proposed method, we performed segmentation on the WBCS and RBCs separately and developed distinct preprocessing steps for each. This step helped us to obtain more accurate results, where as they implemented the same preprocessing for both cell types, which were not separated. They used the *b* channel for WBCs and the *L* channel for RBCs. Three sample images were used to develop general parameter values for CIELAB, which was used to filter the cells. Only three sample images were tested to justify the thresholding values, which are insufficient to generalize for all images. Hence, it leads to some information loss, which affected their performance. Unlike basic CHT, our proposed method was able to detect a variable degree of overlapping and irregular cells.

Putzu and di Ruberto [28] used the same data set and presented a method that only detected and counted WBCs. They selected 33 images to test. The watershed technique was used to segment and separate overlapping cells and the average accuracy for detecting and counting the cells in the 33 images was 93.5%.  Table 3 compares the accuracy of our proposed method to other methods.

**Figure 22:**
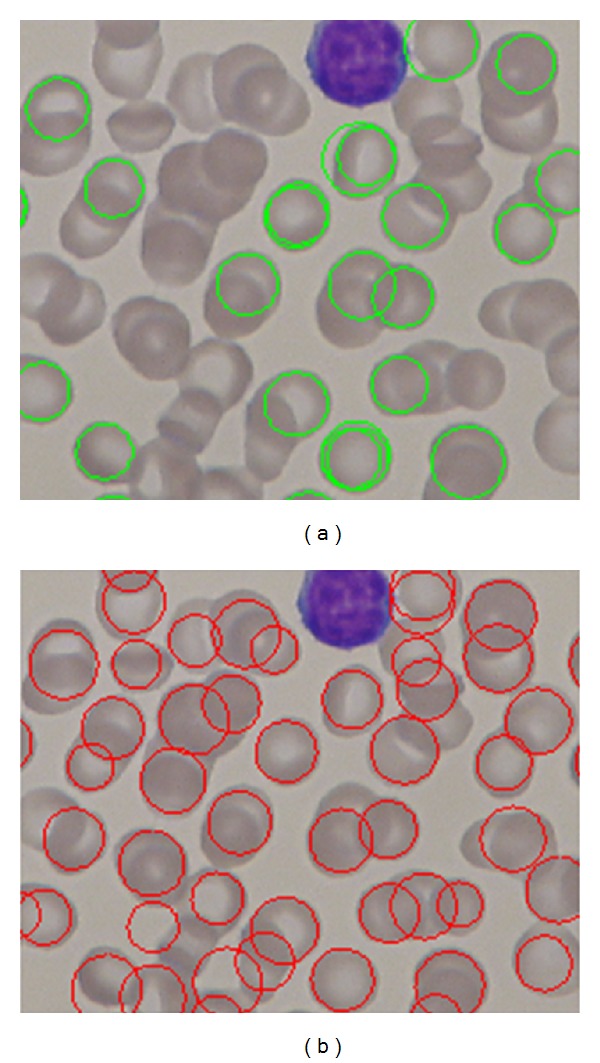
(a) CHT RBCs detection results and (b) our proposed method RBCs detection results in overlapping cells.

Furthermore, we also have conducted similar experiment using the same dataset and the same preprocessing steps using the classic circular Hough Transform CHT [19] to detect RBCs. Our findings justified that our proposed method outperforms the classic CHT. The comparison results can be shown in Table 3; the basic CHT achieves 67% average accuracy for detecting RBCs, whereas our proposed method achieves 97.5%. Our proposed method has shown better results in sensitivity (PR) and specificity (RC). With the introduction of iterative structured circle detection algorithm, our proposed method is able to detect irregular cells and tolerate with a certain degree of overlapping cells, which it leads to produce higher probability to detect more cells that are correct. Unlike our proposed method, basic CHT method gains slightly lower performance when detecting overlapping cells as shown in Figure 22.

**Figure 23:**
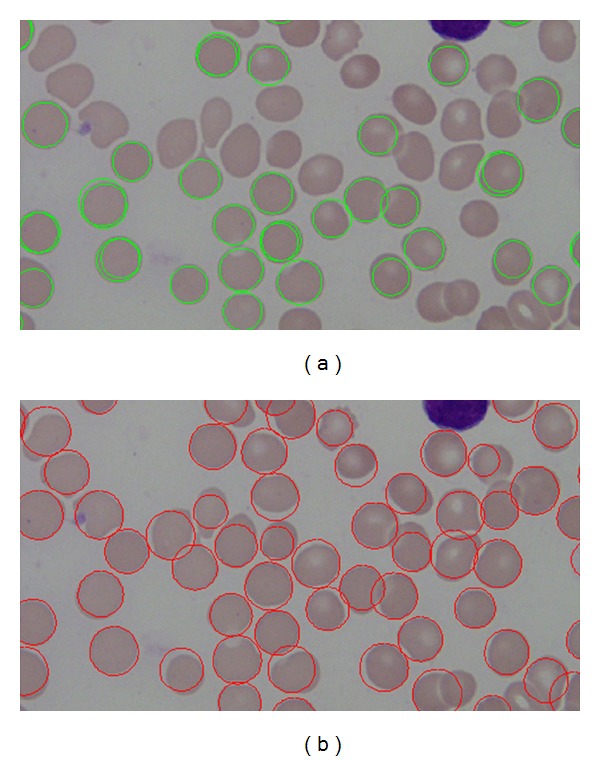
(a) CHT performance when detecting irregular cells and (b) the proposed method performance when detecting irregular cells.

In case of other cases irregular cells, our proposed method presents a better performance than CHT in that case as shown in Figure 23.

## 5. Conclusions

This paper proposed a method to automate the segmentation and counting of red and white blood cells using iterative structured circle detection algorithm. Several improvements were made to the RCD algorithm, including an initialization step to find 8-neighbor connected component. Additionally, the proposed model features an enhanced probability of selecting the correct circle from four candidate circles, the capability to detect irregular cells, the use of dynamic number of iterations, and improved detection of overlapping cells. The proposed method performed the segmentation and counting of WBCs and RBCs well when results were compared with the ground truth, which was determined by experts. The following segmentation and counting accuracies were achieved using the proposed method: PR = 89.7%, RC = 98.4%, and *F*-measure = 93.9% for WBC and PR = 95.3%, RC = 97.5%, and *F*-measure = 96.4% for RBCs.

## Figures and Tables

**Table 1:** Summary of results for WBCs.

Set	Manual count	Proposed method count	TP	FN	FP	PR	RC	FM
1	117	118	116	1	2	98.3%	99.1%	98.7%
2	192	199	191	1	8	95.9%	99.4%	97.6%
3	53	57	53	0	4	92.2%	100%	96.3%
4	15	13	13	2	0	100%	86.6%	92.8%
5	99	108	95	4	13	87.9%	95.9%	91.7%
6	298	350	297	1	53	84.8%	99.6%	91.6%
7	73	82	72	1	10	87.8%	98.6%	92.9%
8	28	31	26	2	5	83.8%	92.8%	88.1%
9	41	47	40	1	7	85.1%	97.5%	90.9%
10	34	37	32	2	5	86.4%	94.1%	90.1%

Total	950	1042	935	15	107	90%	98%	94%

**Table 2:** Summary of results for RBCs.

Set	Manual count	Proposed method count	TP	FN	FP	PR	RC	FM
1	2117	2123	2076	41	47	97.7%	98%	97.9%
2	2553	2586	2474	79	112	95.6%	96.9%	96.2%
3	2484	2529	2443	41	86	96.5%	98.3%	97.4%
4	3788	3799	3689	99	110	97.1%	97.3%	97.2%
5	4899	5107	4798	101	309	93.9%	97.9%	95.9%
6	4887	5133	4699	188	434	91.5%	96.1%	93.7%
7	5195	5228	5007	188	221	95.7%	96.3%	96%
8	4223	4341	4190	33	151	96.5%	99.2%	97.8%
9	4474	4588	4394	80	194	95.7%	98.2%	96.9%
10	4974	5044	4842	132	202	95.9%	97.3%	96.6%

Total	39594	40478	38612	982	1866	95%	98%	96%

**Table 3:** Accuracy of the proposed method compared with other methods.

Method	RBCs	WBCs	RBC's		
	Average accuracy	Average accuracy	PR	RC	FM
Color-based and Hough transform [22]	64%	81%			
Morphology operators and watershed algorithm [28]	—	93.5%			
Basic Circular Hough Transform (CHT)	67%	—	71%	68%	71%
Our proposed method	97.5%	98.4%	95%	98%	96%
